# Understanding Barriers and Facilitators to Online and App Activities for People Living With Dementia and Their Supporters

**DOI:** 10.1177/08919887221149139

**Published:** 2023-01-04

**Authors:** Abigail R. Lee, Orii McDermott, Martin Orrell

**Affiliations:** 1 Mental Health and Clinical Neurosciences, Institute of Mental Health, 170718 University of Nottingham, Nottingham, UK

**Keywords:** dementia, independence, questionnaire, quality of life, computer technology, online

## Abstract

**Background:**

Stigma often surrounds people with dementia when it comes to use of computer technology, although evidence does not always support this. More understanding is needed to investigate attitudes and experience in relation to computer technology use among those living with dementia and their readiness to use it to support self-management.

**Methods:**

An online self-report questionnaire was completed by adults living with a dementia diagnosis and those living with them. Questions explored how long the participants had been using computer technology; how regularly they used it; the popularity of common communication apps; and whether they were interested in using an app to support their independence.

**Results:**

47 participants with dementia and 62 supporters responded to the questionnaire. There were no obvious differences between those with dementia and supporters when it came to regular technology usage and both groups showed positive attitudes to the use of it for independence in dementia.

**Conclusions:**

There was active use of computer technology among this population. Benefits were shown to include communication, increasing individuals’ understanding of dementia diagnoses, and enabling independent activities for both those with dementia and supporters.

## Introduction

In the past, people living with dementia have often been stigmatised as passive consumers of technology, who rely on their supporters to facilitate use.^1^ However, technology can support people with dementia to stay engaged with meaningful activities, access resources, maintain independent relationships and achieve a decent quality of life.^2^ Having a better understanding of the digital skills, confidence in and use of technology would help improve the guidance and inclusion for improving access to technology in people with dementia.^2^ The increase in available technology, in particular computer technology such as smartphones and tablets, has raised questions as to how they could be used in dementia care services and in the community to support families and improve the wellbeing of those living with dementia.^3^ Little is known about how familiar people living with dementia are with technology and how often they use it. With the Covid-19 pandemic, many people living with dementia and their families have become more socially isolated and lost participation in meaningful activities.^4^ Technology has enabled people to access services, resources and support during the social restrictions and closure of many healthcare services.

Existing literature shows a mixed reaction to and use of technology in people living with dementia. Adults with subjective cognitive impairment (SCI), mild cognitive impairment (MCI) and dementia were surveyed about their use of technology and interests in electronic health (eHealth).^5^ Most people were routinely using mobile phones and computers, had access to internet in their own homes, and experience in emails but were unfamiliar with social media. Although only a one in 10 of the sample had dementia, this study revealed that use of technology is prevalent in people with varying cognitive difficulties, raising the possibility that eHealth interventions could be of interest to some within this population, with the right support available. A similar study surveyed older adults living with MCI or mild dementia.^6^ A quarter used smartphones almost daily, with many using specific applications to support their memory. Participants found to have higher enthusiasm for technology showed fewer depressive symptoms and a better health status score. Findings from this study revealed that attitudes to and daily use of technology varies among people with cognitive conditions and their supporters, but that there could be potential for it having a positive impact of depressive symptoms and health. However, only 27% had dementia so the findings are difficult to generalise.

The RE-AIM Study of the PRIDE Self-Management App is exploring the reach, adoption, and potential effectiveness of an online handbook for people living with mild dementia to help them improve their independence by helping them to productively use their time and activities. Online interventions need to understand their target audience. In our PRIDE-app study,^7^ recruitment was slow compared to the study using the PRIDE paper-based manual which recruited over 90 people.^8^ Although the evidence suggests that technology could play a role in dementia support, the adoption of such interventions is reliant upon people with dementia accepting it and feeling engaged. Technology acceptance relates to the attitudinal perception and behavioural intention to use technology and has a significant role in predicting whether technology will be adopted and used.^9^ However, to assess how far people with dementia are ready to use apps for self-management and independence, we need to know more about their internet use and approach to a variety of technologies. The aim of the study was to investigate attitudes and experience in relation to computer and smartphone technology use among those living with dementia and their readiness to use it to support self-management.

## Methods

### Participants

Any adult aged 18 years or over who either had dementia or lived with/supported someone with dementia were eligible to complete the questionnaire. A minimum target of 80 participants and a maximum of 250 was set, with these figures taking in both people with dementia and supporters. This target was set due to the short period of time the questionnaire was available for and considered that advertisement of the survey was reliant upon participants receiving notifications, as mentioned below.

To recruit participants, the link to the questionnaire was posted on the Join Dementia Research (JDR) website. This is an online self-registration service that enables volunteers with memory problems or dementia, carers of those with memory problems or dementia, and healthy volunteers to register their interest in taking part in research. The inclusion criteria were for participants with dementia to have a medically confirmed diagnosis of any form of dementia and live in the community were set. Inclusion for supporter/carers only required them to be an active supporter of someone with dementia who was living in the community. The volunteers signed up to JDR received an alert about the study if they meet the inclusion criteria. Then, eligible participants had the opportunity to access the questionnaire and contact the study team if they had any questions. A paper version of the questionnaire was available to request and could be posted to participants with a prepaid return envelope. The questionnaire link was live for 6 weeks from April to June 2022.

### Development of the attitudes Towards Technology Questionnaire

Following a scope of the research and defining the research questions, a 28-item self-report questionnaire was created using the JISC online platform (formally known as Bristol Online Surveys), an online tool designed for researchers which allowed participants to easily access the questionnaire. This platform enabled us to create questions, decide on the answer response (e.g., whether multiple answers could be selected), and select which were required and which were optional questions. There were also six demographic-based questions to record whether the participant was living with dementia or a carer/supporter, along with their age, gender etc. Questions were developed by scoping similar surveys published online^10,11^ and through several rounds of revisions following discussions within the research team prior to the final version going live. This was to try and maximise the relevance and cover of questions in terms of the study aim.

Questions explored how long the participants had been using computer technology; how regularly they used it; the popularity of common communication apps; and whether they were interested in using an app to support their independence. For this questionnaire, computer technology was identified as computers, laptops, tablet computers and smartphones. Most questions had a “Yes” or “No” answer, or a range such as “Very knowledgeable” to “Not at all knowledgeable”. Several questions encouraged respondents to expand on their “Yes/No” answer by providing qualitative data. Many models and questionnaires have been created to predict technology acceptance, including the TechPH questionnaire,^10^ which was incorporated into the present survey. The six TechPH questions included were answered using a five-point Likert scale, with response options ranging from “Fully disagree”(1) to “Fully agree”(5). The final version of the questionnaire can be found in Supplemental Appendix 1.

### Ethics

This sub-study was reviewed and given ethical approval by Oxford Research Ethics Committee (21/SC/0066). All minor and substantial amendments were reviewed by the University of Nottingham before being approved by the Oxford Research Ethics Committee. Consent was gathered on the information page, where all participants had to tick the consent box to proceed.

### Analysis

Data was imported into SPSS 28 for analysis and checked and cleaned. Although two respondents did not provide an answer, after reviewing the data during cleaning, one provided another answer which indicated that they were in the supporter role and was added to that group. The other respondent indicated that they were neither living with a dementia diagnosis nor were a supporter of someone who was. Therefore, their data was removed from the dataset prior to analysis. Qualitative data from all participants was reviewed and grouped together with the corresponding quantitative question, to enhance the understanding of the numerical figures.

## Results

### Participant Demographics

Table 1 presents the summary demographic data for the questionnaire respondents. Of 110 participants, 61 (55.5%) were supporters and 47 (42.7%) were people with a dementia diagnosis. In the 47 participants with dementia, time since diagnosis ranged from six weeks to 23 years. Most completed the questionnaire alone (n = 40, 85.1%) and seven participants reported having assistance (14.9%). There were considerably more male respondents (n = 34, 72.3%) than female (n = 12, 25.5%). With regards to their ages, 80.8% of participants were between 65 and 84 years old (n = 38), and 97.9% were of White ethnicity (n = 46). 34% (n = 16) had completed High School and 46.8% (n = 22) held a degree. one participant did not provide an answer to the demographic questions.

**Table 1.** Summary Statistics for Demographic Details of Participants.

Demographics	People with dementia (n = 47)	Supporters (n = 62)
Time since diagnosis/Duration of informal caregiver	Six weeks – 23 years (M = 4.5 years, SD = 4.44)	Six weeks – 30 years (M = 5.6 years, SD = 4.85)
Age	45 – 64 years 14.9% (n = 7)	18 – 44 years 3.2% (n = 2)
	65 – 74 years 31.9% (n = 15)	45 – 64 years 50% (n = 31)
	75 – 84 years 48.9% (n = 23)	65 – 74 years 30.6% (n = 19)
	85+ years 2.1% (n = 1)	75 – 84 years 14.5% (n = 9)
		85+ years 1.6% (n = 1)
Gender	Male 72.3% (n = 34)	Male 14.5% (n = 9)
Ethnicity	White 97.9% (n = 46)	White 93.5% (n = 58)
Highest education Level (high school or above)	Degree 46.8% (n = 22)	Degree 50% (n = 31)
Use(d) computer technology for work	Yes 76.6% (n = 36)	Yes 79% (n = 49)
Internet access from home	Yes 97.9% (n = 46)	100%
Time of length using computer technology	2+ years 95.7% (n = 45)	2+ years 96.8% (n = 60)
Main use for technology	Email/communication 89.6% (n = 42)	Email/communication 96.8% (n = 60)

The 62 supporters had been in their roles for vastly varying durations, from six weeks to 30 years. The majority were aged between 45 and 74 years old (n = 50, 80.6%). As expected from experiences with the main study recruitment, almost all supporters were of white ethnicity. There were two from Asian/Asian British ethnicity (3.2%), and one from Black/African/Caribbean/Black British ethnicity (1.6%). The question about the highest level of education reached revealed that 33.9% (n = 21) had completed High School and 50% (n = 31) had completed a degree.

A vast majority were found to have been using computer technology for two or more years, which indicated most were regularly using it prior to the COVID-19 pandemic, rather than learning to use it during. Most supporters had also been using technology for two or more years. Much like the figures from people living with dementia, this shows that technology use among supporters was popular prior to the COVID-19 pandemic.

Email and communication were the most popular use for computer technology in both groups, with the news and weather (n = 36, 76.6%) and shopping (n = 29, 66%) also proving popular for people with dementia. As communication apps, such as WhatsApp and Zoom, were both used by almost 80% of this group (n = 37, 78.7%), this would support the most common use of computer technology.

### Internet Use

Table 2 details the internet, email, and general computer technology use amongst the participants. Use of the internet was daily or almost daily for 89.4% (n = 42) of people with dementia, with emails being accessed either every day or almost every day for 36 (76.6%) respondents. When asked if they had used any computer technology in the month preceding the questionnaire, 61.7% (n = 29) reported using it daily and a further 25.5% (n = 12) almost every day. A Chi-Square was conducted on internet use and time since diagnosis (less than/equal to 2 years or more than 2 years), which were not found to be related (*X*^2^ (1, *N =* 45) = 1.6, *P = .*254). As the result was not significant, it revealed that Those diagnosed more than two years ago were just as likely to use the internet daily as those diagnosed under 2 years ago.

**Table 2.** Summary of Usage Data Reported by Respondents.

Usage	People with dementia (n = 47) (*Every day/Almost every day*)	Supporters (n = 62) (*Every day/Almost every day*)
Internet use	89.4% (n = 42)	96.8% (n = 60)
Email use	76.6% (n = 36)	92% (n = 57)
Use of computer technology in past month	87.2% (n = 41)	96.8% (n = 60)

### Views on Technology

Table 3 presents the participants’ views on technology and how knowledgeable they consider themselves. Over 60% of people with dementia said it was fun to learn how to use new technology, and most felt it made life easier, but there was also an agreement that the technological progress of today was hard to keep up with. The percentage of people with dementia who liked to acquire the latest technology indicated that this group presented as more innovative and interested in having new technology. A Chi-Square on knowledge level and acquiring the latest models showed a significant relationship between groups (*X*^2^ (20, *N* = 62) = 65.6), *P* = .001). With regards to supporters, the enjoyment of learning how to use new technology was also over 60%. Parallels between groups were revealed with regards to their enjoyment of new technology, the positive impact it had on making lives easier and in agreement that the technological progress was hard to keep up with.

**Table 3.** Summary of Respondents’ Views on Technology.

Views on Technology (statements)	People with dementia (n = 47) (Fully agree/agree)	Supporters (n = 62) (Fully agree/agree)
It’s fun learning how to use new technological gadgets	62% (n = 29)	61% (n = 38)
Using technology makes life easier	89% (n = 42)	89% (n = 55)
I like to acquire the latest models or updates	47% (n = 22)	24% (n = 15)
Today, the technological progress is so fast that it’s hard to keep up	72% (n = 34)	82% (n = 51)
	People with dementia (n = 47) (Very/Quite knowledgeable)	Supporters (n = 62) (Very/Quite knowledgeable)
1. How knowledgeable do you consider 2. yourself to be when it comes to using a 3. computer, tablet, or smartphone?	53.2% (n = 25)	72.6% (n = 45)

Table 4 provides an overview of the qualitative feedback gathered from both group of participants. Figures and quotes demonstrate the lack of difference between groups, with people with dementia and supporters both experiencing apprehension when using new technology. Over half of both groups shared that they had apprehension for using new technology, with the sometimes-complex nature of technologies being identified as a main anxiety. For those with dementia who did not share this uncertainty, their reasoning showed determination and persistence to not allow their age or dementia to deter them from learning about new technology.

**Table 4.** Views of Participants.

Views on Technology (statements)	People with dementia (n = 47) Fully agree/agree	Example quotes from people with dementia	Supporters (n = 62) Fully agree/agree	Example quotes from supporters
Sometimes I’m afraid of not being able to use the new technical things	66% (n = 31)	*“Difficult to understand how to use.”* *“Need lots of repeated support…to grasp the new tech.”*	56% (n = 35)	*“I struggle if new software/technology goes wrong.”* *“They [computer technologies] can be quite complicated.”*
I would have tried new technical gadgets to a greater extent if I had more support and help than I have today	38% (n = 18)	*“Need lots of support to understand how a new gadget could be helpful.”* *“My family lose patience when teaching me how to use technology.”*	36% (n = 22)	*“Don’t know implications and how to rectify problems.”*

### Technology for Dementia

One area of great interest to us was whether computer technology is already widely used by people living with dementia to access resources and support. Table 5 provides a quantitative and qualitative overview of the current uses of computer technology for dementia support among participants, as well as their concerns and priorities for technology. The majority accessed national and local dementia groups websites, such as the Alzheimer’s Society; attended virtual support groups for those diagnosed; researched symptoms; and a number were receiving notifications of new research and journal publications. Existing use of computer technology to support their independence and daily activities included alarms and reminders; communication with family and friends; and electronic diaries to keep track of appointments and medication. Through their comments, it was clear to see that computer technology enabled the independence of people with dementia as a source of additional support. The four most common concerns were data protection and privacy; security and fraudulent activity; a lack of knowledge; and making mistakes, including deleting important information and accidentally sharing personal information. These concerns were reiterated to an extent when respondents were asked whether they had any priorities for future computer technology.

**Table 5.** Use of Technology for Supporting People With Lived Experience of Dementia in Their Daily Lives.

Technology for dementia	People with dementia (n = 47)	Example quotes from people with dementia	Supporters (n = 62)	Example quotes from supporters
Knowledge level – very or quite knowledgeable	53.2% (n = 25)		72.6% (n = 45)	
Already accessing dementia-related resources	Yes 72.3% (n = 34)	*“Medical information, diet advice. Lifestyle advice and academic studies.”* *“I use technology to do research on Alzheimer’s and to connect what Alzheimer’s groups and organizations.”*	Yes 79% (n = 49)	*“Research info on symptom developments.”* *“Online forums for carers.”* *“Alzheimer’s Society, Alzheimer’s Research, browsing latest research etc.”*
Use for daily activities and independence	Yes 72.3% (n = 34)	*“I depend on my computer, tablet, and smartphone for most things.”* *“Researching my condition including any new symptoms.”* *“Receiving counselling and support via teams/Zoom.”*	Yes 53.2% (n = 33)	*“Helps me stay in touch and manage my own life alongside the demands of being full time carer.”* *“My smartphone is my link to my own life…I don’t think I could do this caring role without the support I get via my phone.”*
Highlighted concerns	• Data protection • Security • Lack of knowledge • Making mistakes	*“Lack of knowledge and fear of pressing the wrong button.”* *“Data protection and identity theft.”*	• Security • Fraud • Lack of knowledge	*“…worries around security, personal information being used.”* *“Not knowing what I am doing. Feeling frustrated when I can’t get it to work.”*
Priorities for future technology	• Easily accessible • Simple to use • Secure	*“Data protection and minimizing scams is very important.”* *“Simple to use, easy to understand.”* *“Support with remembering all the passwords etc. in easy accessible way.”*	• Accessible • Simplicity • Secure	*“Easily accessible, interesting to use, helpful to my situation.”* *“User-friendly, safe, ethical.”* *“Simplicity, works for person with dementia to use or can be remotely used to support them.”*

When asked about the use of an app to support their daily activities, the 72.3% (n = 34) who were interested provided insightful reasoning as to why from the perspective of living with a diagnosis:

*“Anything that helps myself and wife to deal with this [dementia].”* [Person with dementia]

*“Anything that could be of benefit to me of make my life easier.”* [Person with dementia]

*“Help keep my mind active.”* [Person with dementia]

*“I realise there is a lot of potential to be more self-sufficient.”* [Person with dementia]

Accessing dementia-related content was popular among most supporters, with similar resources accessed to people with dementia, such as charity website; support groups for informal carers; dementia-specific training courses; and as a source of symptom information. Through their comments, it was evident that computer technology provided a lifeline for supporters as a source of additional support and highlighted the benefits of online resources. As expected, supporters shared similar concerns about computer technology as those with dementia, with security, fraudulent activity and a lack of knowledge frequently reported. Supporters’ priorities were very similar to people with dementia, with accessibility and ease of use reported by many.

As supporters reported many similar concerns and priorities for computer technology, it shows shared viewpoints among both groups. It perhaps reinforces the argument for including people with dementia in the development stages of relevant technology, as they are aware and have a good understanding of the potential benefits and pitfalls and are good at communicating what they want out of technology. Supporters were also asked whether they would be interested in using an app regularly on their computer, tablet, or phone to support their independence and daily activities. Understandably, and much like the responses from people with dementia, there were some supporters who did not feel that their situations would benefit from this, or that it would be easy to use regularly (n = 28, 45.2%). However, for the small majority who did say ‘Yes’ (n = 33, 53.2%), their reasoning centred around supporting their relatives and enabling their independence:

*“Anything that can help my sister be as independent as possible but also safe.”* [Supporter]

*“Anything that helps me to manage my situation.”* [Supporter]

*“Anything to help independence is good.”* [Supporter]

*“If it was something that helped my husband maintain his independence I would be interested.”* [Supporter]

*“I’m always looking for resources that will help me provide a better quality of life for my wife.”* [Supporter]

## Discussion

### Principal Findings

People with dementia and supporters actively used computer technology for a variety of needs and showed an interest in maximising its use to support independence in those living with the condition. To our knowledge, this questionnaire provides the first comparison of attitudes towards the daily use of general computer technology in people living with dementia and supporters. There were no obvious differences found between groups in terms of their usage and range of computer technology use, but the majority of those with dementia were older males, whereas supporters tended to be younger females. Contrary to the common myth that older adults did not use computer technology, 51% of respondents with dementia were aged 75 and over. The broad ages of supporters, and the higher number of females, would suggest that there were more daughters and wives in this role. This is in accordance with what has been found globally with regards to informal care in dementia.^12^ The survey figures question why there was a gender divide within the people with dementia group, and whether females with dementia were less willing to use computer technology, less interested in completing surveys, or if they simply did not have the time to answer the questionnaire. As the ages of participants include those of normal working age, it is reasonable to infer that some participants were likely to currently be using computer technology for work. When interpreting the findings, it should be considered that the participant population were heavily white British people.

Communication was identified as a primary use for computer technology, which demonstrates the importance of reducing social isolation and maintaining relationships for people living with dementia. Technology enjoyment level among supporters showed similarity to the attitudes of those with dementia, which was not expected due to the nature of the condition. As anticipated in a sample of older adults with cognitive difficulties, apprehension for using technology was shared by a number of respondents with dementia. Additionally, people with dementia voiced concerns about the use of computer technology, such as security and system issues, which could be indicative of why there is an apprehension to adopt new technology. As over half of supporters also reported apprehension, and a third felt they would have tried more with technology if they had more support, their hesitance or anxiety about using technology could have wider implications for its use among some people with dementia. People with dementia can use technology, but some may require additional support from family or friends in order to feel more confident. Therefore, supporters’ attitudes could, for example, restrict the reach and use of technology within the population of those living with dementia. It was interesting that those who did not share this apprehension placed emphasis on not allowing their age or diagnosis to impede their usage. Knowledge levels in those with dementia appeared to support the apprehension found in using new technology, suggests a potential link between knowledge levels and adoption and that this is an important factor in the confidence and potential adoption of computer technology. Accessing dementia-related resources was popular among the sample, and computer technology provided a majority assistance with daily activities, enabling their independence. Perhaps unexpectedly was the frequent use of computer technology amongst those with dementia and how long they had been using it. As a majority were familiar with computer technology prior to COVID-19, the restrictions and closures during the pandemic may have increased their frequency and range of use rather than prompt learning to use them.

Supporters showed similar computer technology use as those with dementia, with daily use reported amongst the majority of the sample, and communication appeared equally important to them. Just over half reported apprehension with innovative technology, although higher knowledge levels were recorded. This could be interpreted that although they may have the knowledge, supporters sometimes lack confidence in using computer technology, much like those living with dementia. Accessing dementia-related content was popular among many supporters, with similar resources accessed to people with dementia, such as charity website; support groups for informal carers; dementia-specific training courses; and as a source of symptom information. This suggests that some supporters actively seek dementia resources and are keen on improving the informal care they provide. Through their comments, it was evident that computer technology provided a lifeline for supporters as a source of additional support and highlighted the benefits of online resources. It would appear computer technology provides supporters with a valuable link to others in similar roles, resources to improve their support of the person with dementia, and a sense of their own lives outside of caring.

### Methodological Problems

Nearly all the survey questions were interpreted as intended, but during data cleaning one was found to have been ambiguous and their answers influenced by this. When asked if they were living with a dementia diagnosis, meant as having received a diagnosis themselves, several supporters answered ‘Yes’. As there was not the time available to trial the questionnaire beforehand, the study acted as the pilot and changes can be made to reduce the ambiguity of questions for future use from these findings. The majority of respondents being of white British ethnicity was perhaps expected from the researchers’ previous experiences, but the demographic data was still disappointing in terms of diversity. As JDR encourages those from all backgrounds to take part in research, it was hoped that the platform would have increased diversity in the sample. However, more consideration would need to be made for similar surveys in how to widen participation.

### Comparison with Previous Work

Previous evidence has suggested the potential positive impact of technology on people living with dementia and their network, a finding which our survey supports. The current study highlighted regular use of the internet and computer technology among those living with dementia and a generally positive attitude towards it. Previous evidence supports this,^6^ with the study suggesting technology could have a positive effect on people with mild dementia and interest was high in using technology to help the self-management of conditions. Other evidence^5^ found that those with dementia were more likely to experience difficulties when using computer technology due to factors including their age. However, although use varied depending on the factors and diagnoses, most participants used technology routinely, regardless of these factors. Our findings that time since diagnosis did not negatively impact technology use among participants, nor was there a significant difference in internet use between respondent groups go well with LaMonica et al.’s^5^ conclusions in promoting a positive image of technology use among the dementia population. A systematic review of 48 studies explored the barriers and facilitators of assistive technology on older adults with dementia.^13^ Important facilitators identified included the potential to enable independence in dementia and the want from supporters to use the technology. Similarly, the present findings showed enabling independence was a key influence in what people with dementia look for and use technology for. As a contrast however, the study found that people with dementia showed similar enthusiasm for technology, and this would suggest that the role of supporters in wanting computer technology was not as prevalent or pivotal as it was in previous reviews.^13^ Cost and the person with dementia not wanting to engage with technology were the top barriers reported in Kruse et al.,^13^ contrary to our findings of security and anxiety around technical problems.

As a tie into the RE-AIM Study of the PRIDE Self-management App,^7^ respondents were asked whether they would be interested in using an app regularly on their computer, tablet, or phone to support their independence and daily activities. Only 21.3% of people with dementia did not show any interest in using an app to support their activities. Those who were interested were enthusiastic to trial anything that could benefit their independence and help themselves and families come to terms with the diagnosis. With the difficulties experienced in recruitment for the Lee et al. study, the questionnaire results could suggest a gap between the attitudes towards technology in those with dementia, and their behaviour. It would appear that they believe computer technology could benefit their lives and have positive attitudes towards its inclusion and have been using it for dementia-related resources prior to the COVID-19 social restrictions. If this population do engage with technology, as the survey and previous evidence would suggest, then their access to dementia-related technology and research needs to be improved.

### Limitations

Given that the questionnaire received 110 responses, of which the slight majority were supporters of those with dementia, the findings are limited in their generalisability to the dementia population in terms of figures and with regards to the use of JDR as the recruitment platform. Nevertheless, the priorities and concerns for computer technology that were identified are important and necessary considerations for the development of any dementia-specific technology or when widening accessibility to computer technology. Additionally, due to the recruitment platform used, all respondents would have had some previous use of computer technology. However, the broad range in the age and time since diagnosis suggest that the sample comprises people at different stages of dementia progression (and most likely levels of cognitive impairment), making it fairly representative, even if it is skewed in terms of prior technological experience. To improve adoption of technology to support dementia, research is needed within those communities who have minimal experience to understand their attitudes towards it. As the survey did not ask participants whether they were currently employed, we are unable to differentiate between those who previously used technology for work, and those who still do. However, as several participants were within the working age group, it is possible that some were still employed. The questionnaire did not differentiate between distinct types of dementia. Given the variety of types of dementia and the predominant symptoms of each, it would be valuable to understand differences in technology use across dementia type.

### Recommendations for Future Research

To improve adoption of technology to support dementia, research is needed within those communities who have minimal experience to understand their attitudes towards it. Studies should consider the role of digital poverty within communities and how this currently, or will, affect dementia populations. Although researchers and policy makers were aware of the pre-existing digital divide between age groups, especially impacting those living with dementia,^14^ the COVID-19 pandemic emphasized the barriers individuals have which prevent them from utilizing technology.^15^ These included a restricted access to technology, limited knowledge, and a lack of dementia-friendly digital interventions.^14^ Future studies need to prioritse enabling digital skills and technology accessibility within these populations that are being prevented from accessing online resources and digital interventions. In addition, the questionnaire unearthed concerns with security, data privacy, and making significant errors from people living with dementia and supporters. These should be considered when developing digital dementia interventions and other technology, alongside the priorities identified such as easy access, simple to use and secure. Our sample were well educated, mainly White, and had previous experience with computer technology. As these factors are likely to have influenced our findings and do not give us a comparison to the wider dementia population, future surveys should include a diverse sample of people with dementia and their carers to give a more representative view. Additionally, detailing peoples’ specific dementia diagnoses would help explore the differences in technology facilitators and barriers based on dementia type. A comparison between those with dementia and those without (who are not informal supporters) would increase our understanding how attitudes in dementia when compared to the general population. This could be done through an epidemiological and questionnaire study, by gathering views from a representative cross-section of society.

## Conclusion

Contrary to the stigma associated with older adults being reluctant and unable to adopt technology, this study has contributed to raising awareness of the active use of computer technology among this population including those with dementia. Benefits of using computer technology were shown to include communication, increasing individuals’ understanding of dementia diagnoses, and enabling independent activities for both those with dementia and supporters. There were no obvious differences between those with dementia and supporters when it came to regular technology usage and both groups showed positive attitudes to the use of it for independence in dementia. The accessing of dementia-related content helped respondents to better understand a diagnosis and educate themselves further on dementia and its symptoms, as well as connecting the wider dementia community. Although responses were mixed to the use of an app for independence and daily activities, there appeared to be interest in it and enthusiasm about its potential to enable independence among those with dementia. As there appeared to be no disparity between people with dementia and supporters in terms of internet use, the findings demonstrate that individuals with dementia are keen on using computer technology independently and do not usually rely on supporters to facilitate their use. Dispelling the myths around older adults, dementia and technology enables more opportunities to promote adoption and use of computer technology to benefit dementia and improve wellbeing and quality of life, as well as the social aspect which has been shown to be a priority for those diagnosed and their supporters.

## Supplemental Material

Supplemental material - Understanding Barriers and Facilitators to Online and App Activities for People Living With Dementia and Their Supporters Click here for additional data file. Supplemental material for Understanding Barriers and Facilitators to Online and App Activities for People Living With Dementia and Their Supporters by Abigail R. Lee, Orii McDermott, and Martin Orrell in Journal of Geriatric Psychiatry and Neurology
