# Supplementary material for: Understanding Barriers and Facilitators to Online and App Activities for People Living With Dementia and Their Supporters
Source: J Geriatr Psychiatry Neurol. 2023 Jan 4;36(5):366–75. doi: 10.1177/08919887221149139 (PMC10394950; doi:10.1177/08919887221149139)
Supplement: Supplemental material - Understanding Barriers and Facilitators to Online and App Activities for People Living With Dementia and Their Supporters [file sj-pdf-1-jgp-10.1177_08919887221149139.pdf]

Appendix 1

Attitudes towards technology questionnaire

Thank you for your interest in this survey, which explores attitudes towards technology in people living with dementia and their supporters.

Over the last 2 years, during the Covid-19 pandemic, the rate of technology use has accelerated as social contact has become much more restricted at times particularly for many people with dementia. To better understand how technology may continue to be useful to people with dementia and their families, we need to know more about how often they use technology, what types and what they are using it for, plus their attitudes to it. For this questionnaire, the term 'technology' refers to computer technology including computers, laptops, tablets and smartphones.

The PRIDE-app Study is exploring whether an online handbook could support people living with dementia with their independence, making choices and daily activities. PRIDE-app covers information on physical, mental, and social health. Topics include keeping healthy, decision-making, and communication. It encourages the user to set activity plans and make positive changes, both of which can help the user to live well with dementia. You can find out more about the PRIDE-app Study by contacting the research team using the details below.

All of the information collected through this questionnaire will be kept strictly confidential, anonymised and stored securely. If you would like to complete the questionnaire, please complete the consent form and questionnaire. Once finished, please return using the enclosed addressed envelope.

With thanks,

Miss Abigail Lee
PhD Student
University of Nottingham

Email: pride-app@nottingham.ac.uk
Phone: 07890 021703

Attitudes towards technology questionnaire

Are you living with a diagnosis of dementia?
- ☐ Yes
- ☐ No

[If yes] How long has it been since you received your diagnosis?

[If yes] Are you completing this questionnaire by yourself?

Are you currently a carer/supporter of someone living with dementia?
- ☐ Yes
- ☐ No

[If yes] How long have you been in this role?

What is your age?
- ☐ 18 – 44
- ☐ 45 –64
- ☐ 65-74
- ☐ 75 – 84
- ☐ 85+
- ☐ 95+
- ☐ Prefer not to say

What gender do you identify as?
- ☐ Female
- ☐ Male
- ☐ Non-binary
- ☐ Other (please specify)
- ☐ Prefer not to say

Please specify your ethnicity
- ☐ White
- ☐ Mixed/Multiple Ethnic Groups
- ☐ Asian/Asian British
- ☐ Black/African/Caribbean/Black British
- ☐ Other (please specify)

What is the highest level of education you have completed?
- ☐ Some school
- ☐ High School
- ☐ Bachelor’s Degree
- ☐ Master’s Degree

- ☐ PhD or higher
- ☐ Prefer not to say

1. Do you have access to the internet from home?
   - ☐ Yes
   - ☐ No

2. How often do you use the Internet?
   - ☐ Every day
   - ☐ Almost every day
   - ☐ At least once a week, but not every day
   - ☐ Less than once a week
   - ☐ I never use the Internet

3. How often do you use email?
   - ☐ Every day
   - ☐ Almost every day
   - ☐ At least once a week, but not every day
   - ☐ Less than once a week
   - ☐ I never use email

4. On average, how often would you say you have used a computer, tablet or smartphone during the last month?
   - ☐ Every day
   - ☐ Almost every day
   - ☐ At least once a week, but not every day
   - ☐ Less than once a week
   - ☐ Never

5. How long have you been using computer technology, tablets or smartphones?
   - ☐ Less than 6 months
   - ☐ 6 months to 2 years
   - ☐ 2 + years
   - ☐ I do not use them

6. Do you, or did you, regularly use technology for your work?
   - ☐ Yes

     If Yes, then on average how often did you use this technology?

   - ☐ No

7. Do you use any of the following apps?

- ☐ Twitter
- ☐ Facebook
- ☐ Instagram
- ☐ WhatsApp
- ☐ Skype
- ☐ Zoom
- ☐ Microsoft Teams

8. Which of these things do you use technology for?

- ☐ Work
- ☐ Email/Communication
- ☐ Shopping
- ☐ Health
- ☐ News/Weather
- ☐ Games

9. I think it's fun with new technological gadgets

- ☐ Fully agree
- ☐ Agree
- ☐ Don't know
- ☐ Disagree
- ☐ Fully disagree

10. Using technology makes life easier for me

- ☐ Fully agree
- ☐ Agree
- ☐ Don't know
- ☐ Disagree
- ☐ Fully disagree

11. I like to acquire the latest models or updates

- ☐ Fully agree
- ☐ Agree
- ☐ Don't know
- ☐ Disagree
- ☐ Fully disagree

12. I am sometimes afraid of not being able to use the new technical things

- ☐ Fully agree
- ☐ Agree
- ☐ Don't know

- ☐ Disagree
- ☐ Fully disagree

Please could you say a little more why you chose this answer?

13. Today, the technological progress is so fast that it’s hard to keep up

- ☐ Fully agree
- ☐ Agree
- ☐ Don’t know
- ☐ Disagree
- ☐ Fully disagree

14. I would have dared to try new technical gadgets to a greater extent if I had had more support and help than I have today

- ☐ Fully agree
- ☐ Agree
- ☐ Don’t know
- ☐ Disagree
- ☐ Fully disagree

Please could you say a little more why you chose this answer?

15. Do you already use technology to access dementia-related content, resources or support?

- ☐ Yes
  If Yes, please could you expand on this?

- ☐ No

16. How knowledgeable do you consider yourself to be when it comes to using a computer, tablet, or smartphone?

- ☐ Very knowledgeable
- ☐ Quite knowledgeable
- ☐ Not very knowledgeable
- ☐ Not at all knowledgeable

17. Are you currently using a computer, tablet or smartphone in a way to support your independence or daily activities?
    - ☐ Yes

      If Yes, please could you expand on this?

    - ☐ No

18. Would you be interested in using an app regularly on the computer, tablet or smartphone to support your independence and daily activities?
    - ☐ Yes
    - ☐ No

    Please could you expand on your reasons for this?

19. Do you have any concerns about using technology?

20. Do you have any priorities for new technology?

21. Are you interested in knowing more about technology around the house to help you manage with things such as daily tasks and safety?
